# Infestation, community structure, seasonal fluctuation and climate-driven dynamics of mites on small mammals at a focus of scrub typhus in southwest China

**DOI:** 10.3389/fvets.2025.1669217

**Published:** 2025-10-08

**Authors:** Peng-Wu Yin, Yan Lv, Xian-Guo Guo, Wen-Yu Song, Rong Fan, Cheng-Fu Zhao, Zhi-Wei Zhang, Ya-Fei Zhao, Wen-Ge Dong, Dao-Chao Jin

**Affiliations:** ^1^Institute of Pathogens and Vectors, Yunnan Provincial Key Laboratory for Zoonosis Control and Prevention, Dali University, Dali, China; ^2^Institute of Entomology, Guizhou University, Guiyang, China

**Keywords:** chigger mites, gamasid mites, ectoparasites, community ecology, small mammals, seasonal dynamics, generalized additive model (GAM)

## Abstract

**Objective:**

Rodents and other sympatric small mammals serve as reservoir hosts for zoonotic diseases including scrub typhus and hemorrhagic fever with renal syndrome (HFRS), with their ectoparasitic mites (chiggers and gamasid mites) acting as vectors. This 12-month study investigated mite infestation, community structure, seasonal dynamics, and climatic drivers on small mammal hosts in Jingha, southern Yunnan, China–a known scrub typhus and HFRS.

**Methods:**

We calculated infestation metrics (prevalence [*P*_*M*_], mean abundance [*MA*], mean intensity [*MI*], constituent ratio [*Cr*]) and community indices (richness [*R*], Shannon-Wiener diversity [*H*], Pielou evenness [*E*], Simpson dominance [*D*]). Generalized additive models (GAMs) analyzed spatiotemporal and climatic patterns.

**Results:**

From 2,424 small mammal hosts (15 species), we collected 142,471 mites (158 species). Chiggers (109 species, 109,093 individuals) significantly outnumbered gamasid mites (49 species, 33,378 individuals; *P* < 0.001) and showed greater richness (*R* = 9.31 vs. 4.61), diversity (*H* = 2.13 vs. 1.97). Rattus andamanensis was the dominant host. Chigger infestation (*P*_*M*_ = 86.14%, *MA* = 45.01, *MI* = 52.25) significantly exceeded gamasid mites (*P*_*M*_ = 67.16%, *MA* = 13.77, *MI* = 20.50; *P* < 0.001), particularly on female and adult hosts. Four species dominated (*C*_*r*_ = 65.40%): chiggers *Walchia micropelta, Ascoschoengastia indica, Leptotrombidium deliense*, and gamasid mite *Laelaps nuttalli*. Primary vectors among 23 species included chiggers *L. deliense, A. indica, L. scutellare*, and gamasid *Laelaps echidninus* (*C*_*r*_ = 38.46%).

**Conclusion:**

Community indices fluctuated monthly without distinct peaks, while dominant species abundances varied significantly. Climatic factors exerted species-specific effects: *L. deliense* peaked in July (30.0 mites/host; 95% CI: 29.2–30.8) coinciding with maximal temperatures, while *A. indica* peaked in August (25.1 mites/host; 95% CI: 24.5–25.8), lagging peak rainfall. Non-overlapping confidence intervals indicated temporal niche separation between species. Mite-mite networks revealed positive intragroup correlations but no significant intergroup correlations. Host-mite networks demonstrated low host specificity: individual hosts harbored multiple mite species, and individual mite species parasitized multiple hosts. High mite abundance, co-occurrence of multiple vector species, and low host specificity collectively elevate transmission risks and persistence of scrub typhus and HFRS.

## 1 Introduction

The small mammals in this study include rodents (Rodentia) and sympatric species such as shrews and tree shrews (1, 2). They commonly harbor numerous ectoparasites—predominantly fleas, sucking lice, chigger mites, and gamasid mites—with occasional occurrences of ticks. These mammal hosts, alongside their ectoparasitic arthropods, can serve as critical reservoir hosts for zoonotic diseases such as plague, murine typhus, scrub typhus, and hemorrhagic fever with renal syndrome (HFRS) (3–5). Chiggers (chigger mites) and gamasid mites constitute the predominant arachnid groups infesting these hosts. Besides directly inducing dermatitis through their bites, these mites can act as recognized or potential vectors and as reservoir hosts of several zoonotic pathogens. Due to their medical significance, chiggers and gamasid mites are important subjects for epidemiological research (6–9).

Taxonomically classified within subphylum Chelicerata, class Arachnida, and subclass Acari, chiggers and gamasid mites exhibit phylogenetic divergence: chiggers constitute the order Trombidiformes (infraclass Acariformes), whereas gamasid mites belong to order Mesostigmata (superorder Parasitiformes) (10, 11).

Chiggers are the larvae of trombiculid mites (Trombiculidae), which exhibit a seven-stage life cycle, with larvae (chiggers or chigger mites) being the sole ectoparasitic phase. These larvae primarily parasitize rodents and other small mammals and serve as the exclusive vectors for scrub typhus (12–14). *Orientia tsutsugamushi*, the etiological agent of scrub typhus, circulates among rodent reservoirs via chigger bites and may transmit to humans. Certain chigger species (e.g., *Leptotrombidium scutellare*) additionally function as potential vectors for HFRS, which is caused by hantavirus (12, 15–17). These zoonotic diseases pose a significant global health threat, demonstrating rapidly increasing prevalence and expanding endemicity in recent years (18–20). Examples include the recent confirmation of autochthonous scrub typhu transmissions in the United Arab Emirates and Chile, along with suspected pathogen presence in Kenya, despite the disease having been historically confined to the Asia-Pacific region (21–24). The life cycle of gamasid mites comprises eggs, larvae, protonymphs (first nymphal stage), deutonymphs (second nymphal stage), and adult males and females. All developmental stages of gamasid mites can parasitize rodents and other small mammals as ectoparasites. While gamasid mites frequently infest and bite humans, leading to dermatitis, certain species also function as vectors of rickettsial pox and are regarded as potential vectors of HFRS (8, 25–27). Further epidemiological significance associated with gamasid mites on rodents and small mammals is implicated in the transmission of more than 20 additional zoonotic diseases, including endemic typhus (murine typhus), plague, and leptospirosis (28–30).

Yunnan Province in southwestern China represents a significant natural endemic focus for scrub typhus and HFRS. The disease burden is particularly severe in southern regions, exemplified by Xishuangbanna Prefecture (7, 12). This prefecture, bordering northern Myanmar, is a high-risk area for zoonotic diseases including scrub typhus and HFRS, with 1,208 scrub typhus cases were documented between 2006 and 2017. Northern Myanmar similarly experiences outbreaks of scrub typhus and HFRS (15, 17, 31). Mites parasitizing small mammals (e.g., chiggers and gamasid mites) may disperse across borders through host migration, which may facilitate disease transmission. Host migration across international borders elevates zoonotic disease transmission risks, consequently intensifying threats to the growing frequency of international trade and tourism within this region (32, 33). Consequently, the research on small-mammal-associated mites in southern Yunnan's China-Myanmar border region holds critical public health significance.

Both chiggers and gamasid mite groups demonstrate remarkable species diversity, with >3,000 chigger species and >8,000 gamasid mite species documented globally (27, 34, 35). The species-level identification of these mites is taxonomically challenging, requiring slide-mounted specimens for detailed microscopic comparison of morphological characters. The chigger identification proves particularly demanding, necessitating high-magnification (including oil-immersion) microscopy for meticulous observation, morphometric analysis, and comparative assessment—a specialized, labor-intensive process (16, 17, 22). Owing to their diversity, complex identification requirements, and methodological constraints, prior studies have predominantly examined these mite groups separately (6, 7). Despite significant taxonomic divergence (distinct orders/superorders), they frequently co-occur on rodent hosts, forming an integrated ecological mite community. The holistic investigation of this community is therefore warranted.

We conducted monthly surveys at a fixed site in Jingha village, Jinghong County (Xishuangbanna Dai Autonomous Prefecture, southern Yunnan) from April 2016 to March 2017. Using this 12-month dataset, we conceptualize chigger and gamasid mite assemblages on small mammals as a cohesive “mite community.” We characterized its structure and seasonal dynamics while employing Generalized Additive Models (GAMs) to quantify climatic drivers of mite distribution. This integrated approach aims to: (1) advance medical acarology, (2) develop novel methodologies for studying medically significant arthropods and epizoic parasites, and (3) establish scientific foundations for monitoring and controlling vector mites and associated zoonotic diseases in the China-Myanmar bordering region.

## 2 Materials and methods

### 2.1 Field investigations

**Figure 1:**
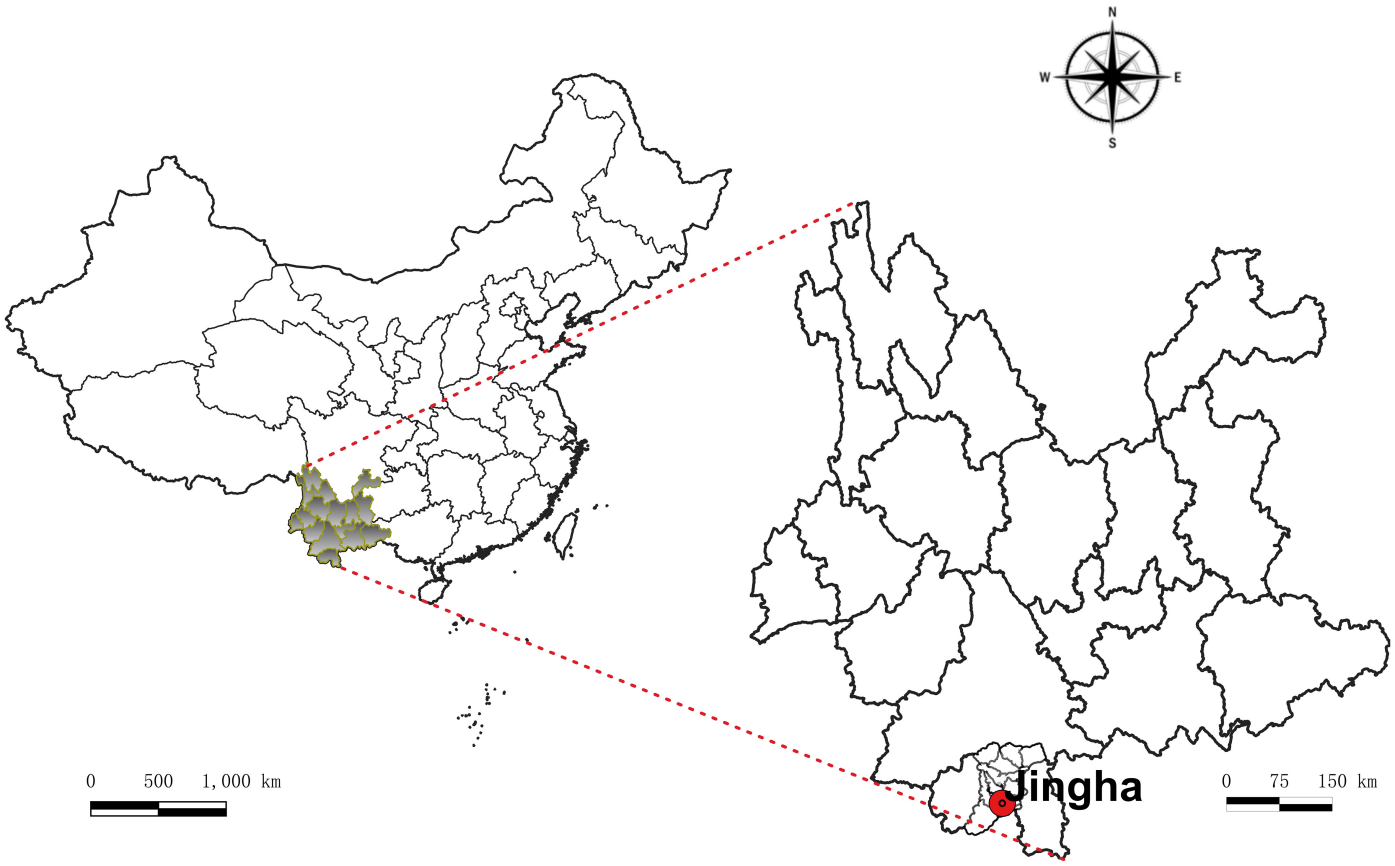
Location map of Jingha survey site in Jinghong County, Xishuangbanna Prefecture, southern Yunnan, China (April 2016–March 2017).

We selected Jingha village, Jinghong County (Xishuangbanna Dai Autonomous Prefecture, southern Yunnan; Figure 1) as a fixed study site. Monthly field surveys (each spanning 15–20 days) were conducted over 12 consecutive months from April 2016 to March 2017. Situated along the Lancang River (Mekong River, flowing northwest-southeast through Yunnan) at 21°50′N, 100°52′E, the study area ranges from 500 to 700 m elevation. This characteristic valley basin landscape encompassed representative habitats including rubber plantations, banana cultivations, farmlands, shrublands, and broadleaf forests (36, 37). Meteorological data were acquired from publicly accessible online records published by regional weather authorities during 2016–2017 (source: https://tianqi.911cha.com/jinghong/).

### 2.2 Collection of mites and host animals

Small mammal hosts (primarily rodents) were captured using baited wire cages (18 × 12 × 9 cm; Guixi Rodent Trap Factory, Guixi, Jiangxi, China). Each host individual was isolated in a separate cotton bag and transported to a field laboratory. Following anesthesia, the mammal hosts underwent examination in a large white tray where chiggers (Trombiculidae) and gamasid mites (Mesostigmata) were systematically collected using standardized parasitological protocols (38). By the help of 10× magnifying lenses, all gamasid mites on the body surface of each host were collected as completely as possible. Due to the microscopic size of chiggers (<0.2 mm), 10× magnifying lenses and surgical scalpels were used to meticulously collect chiggers and chigger-like particulates from hosts' tender skin such as pinnae, auditory canal openings, inguinal regions, perianal areas, and axillae where chiggers often attach. Adhering to a strict “one host to one container” principle, all chiggers and gamasid mites from each host were preserved in 70% ethanol within a pre-labeled centrifuge tube. The hosts were identified to species according to their comprehensive morphology such as size and shape, pelage coloration, morphometrics (body weight, body length, tail length, ear height, hindfoot length), and other morphological features (39–41). Voucher specimens of mites and representative hosts were deposited in the Institute of Pathogens and Vectors, Dali University. The use of animals (including animal euthanasia) for our research was officially approved by the Animals' Ethics Committee of Dali University, approval code: DLXY2001-1116, approval date: 16 November 2001.

### 2.3 Preparation and taxonomic identification of mite specimens

Chiggers, chigger-like particulates and gamasid mites were sorted out and rinsed 2–3 times in clean/distilled water under a stereomicroscope to remove non-mite impurities. Specimens were mounted on glass slides using Hoyer's medium, followed by drying and clearing. Each slide was examined under high-power magnification (100–200×) or oil immersion microscopy (400×), during which morphological structures were meticulously observed, compared, and measured.

According to the hierarchical taxonomic levels (family, subfamily, genus, species), each mite specimen was ultimately identified to species using taxonomic keys and literature (42–44). Specimens unidentifiable to species due to damage, debris obstruction, ambiguous morphology or suspected new species were recorded as species inquirendae (*sp*. *inq*.) in original datasheets. All unidentified specimens were subsequently excluded from statistical analyses. Given the technical complexity of chigger and gamasid mite taxonomy, over 3,000 working hours of dedicated effort were devoted to identification, necessitating significant workload.

### 2.4 Infestation indices and community structure statistics

After taxonomic identification, a verified mite dataset was compiled. Standard statistics of mite parasitism including constituent ratios (*C*_*r*_), prevalence (*P*_*M*_), mean abundance (*MA*), and mean intensity (*MI*) were calculated. Statistical significance tests were conducted with SPSS 26.0 (α = 0.05): Chi-square test for *C*_*r*_ and *P*_*M*_, and Non-parametric test (Mann-Whitney *U* or Kruskal-Wallis *H*) for *MA* and *MI*.


$$\begin{array}{l} C_{r}=\frac{N_{i}}{N}\times 100\%;\;P_{M}=\frac{M_{i}}{M}\times 100\%;\;MA=\frac{N_{i}}{M};\;MI=\frac{N_{i}}{M_{i}} \end{array}$$

In the above formulas, *N*_*i*_ represents the individuals of mite species *i*. *N* represents the total individuals of all mite species. *M* represents the total number of the hosts, *M*_*i*_ represents the number of infested hosts by mite *i*.

Mite community structure was quantified with Margalef richness index (*R*), Shannon-Wiener diversity index (*H*), Pielou's evenness index (*E*), and Simpson's dominance index (*D*). Subcommunity structures of chigger mites and gamasid mites were statistically compared.

### 2.5 Analysis of seasonal dynamics

The corresponding months for each season are as follows: the spring is from March to May, summer from June to August, autumn from September to November, and winter from December to February.

### 2.6 Analysis of climatic drivers for the seasonal dynamics

The overdispersion was corrected by using quasi-Poisson distributions (scale parameters: 1.8–3.2), with Bootstrap test confirming the stability of parameters (consistency > 97%).

### 2.7 Analysis of climate factors driving mite community dynamics

We analyzed climate drivers of mite community distributions on mammal hosts using Generalized Additive Models (GAMs). Models were formulated with the “mgcv” package in R v4.4.3 to fit monthly abundance data of dominant mite species against climate variables (mean temperature, relative humidity, precipitation) (45, 46). A Poisson distribution family and thin-plate regression splines characterized non-linear relationships, with basis dimensions (*k*) fixed at 5 for all smooth terms. Smoothing parameters were optimized via the Unbiased Risk Estimator (UBRE), and 95% confidence intervals (CIs) computed to quantify prediction uncertainty.

(1) Data preprocessing using “tidyverse” package ensure variable validity and model compliance. For each mite species, we specified the GAM as: Mite abundance ~ *s*(mean temperature) + *s*(relative humidity) + *s*(precipitation).

where *s*() denotes a smoothing function capturing non-linear climate-abundance relationships with basis dimension *k* = 5.

(2) Predictions generated via *stats::predict*() (with se.fit = TRUE) enabled confidence interval calculation. Species-specific smooth curves with 95% CIs were visualized using “ggplot2”, faceted by species via *facet_wrap*. Graphical outputs were refined with *theme_publish* and exported as high-resolution figures using *ggplot2::ggsave*().

### 2.8 Analysis of interspecific correlations among dominant mite species

Pearson correlation coefficients were calculated for dominant mite species with larger population sizes within the mite community to evaluate interspecific relationships with a with a significance threshold of α = 0.05. Absolute correlation coefficient (*r*) values were categorized as follows: 0.00–|0.19| (very weak), |0.20|–|0.39| (weak), |0.40|–|0.59| (moderate), and ≥|0.60| (strong).

Correlation matrices and heatmaps generated using Origin 2024 software visually represent relationships among dominant mite species (47).

### 2.9 Analysis of mite-host interactions

Parallel sets diagrams were employed to analyze interactions between mites and their hosts, focusing on dominant mite species with larger population sizes within the community. The analytical workflow comprised the following steps:

(1) The host–mite abundance matrix was converted to long format (host species × mite species). Mite species columns were transformed into attribute columns using Power Query's Unpivot Columns tool to generate a standardized data table.
(2) Parallel sets diagram parameters were configured as follows:
(2.1) Node width: dynamically scaled according to total abundance proportions of hosts and mites using:

$$\begin{array}{l} W_{i}=\frac{Total_{i}}{\max\left(Total\right)}\times 100\% \end{array}$$

where *W*_*i*_ denotes node width and *Total*_*i*_ represents total abundance (host or mite).

(2.2) Ribbon transparency: determined by abundance values, with transparency ranging from 0.2 to 1.0 corresponding to 0–100% abundance:

$$\begin{array}{l} \alpha =0.2+0.8\times \frac{Count_{ij}}{\max\left(Count\;\right)} \end{array}$$

## 3 Results

### 3.1 Species composition and infestations of mites

A total of 2,424 small mammal hosts captured from Jingha survey site (April 2016 to March 2017) were taxonomically classified into 15 species, 10 genera, five families, and three orders (Rodentia, Eulipotyphla, Scandentia; Table 1). *Rattus andamanensis* was the dominant host species, comprising 84.69% (2,053/2,424) of all the hosts. From these hosts, we recorded an amount of 148,958 mite individuals, with 142,471 of which were identified as 158 species, 24 genera, and three families [Trombiculidae (chiggers): 109,093 individuals, 109 species, 12 genera; Laelapidae and Macronyssidae (gamasid mites): 33,378 individuals, 49 species, 12 genera; Table 2]. The unidentified 6,487 mites (4.35%) were excluded from analyses. Among the 158 identified species, 23 (12 chiggers, 11 gamasid mites) were confirmed or potential vectors for scrub typhus or HFRS (Table 2).

**Table 1:** Taxonomic identification of small mammal hosts at Jingha survey site in southern Yunnan of southwest China (April 2016–March 2017).

**Orders**	**Families**	**Number of genera**	**Number of species**	**Individuals**
Rodentia	Muridae	5	10	2,390
	Sciuridae	1	1	8
Eulipotyphla	Soricidae	1	1	1
	Erinaceidae	2	2	11
Scandentia	Tupaiidae	1	1	14
Total	5	10	15	2,424

**Table 2:** Taxonomic identification of mites on small mammal hosts at Jingha survey site in southern Yunnan of southwest China (April 2016–March 2017).

**Taxonomic taxa of mites**	**Identified species and individuals of mites (figures in brackets are corresponding individuals)**
**Chiggers**	A total of 1 family, 12 genera and 109 species with 109,093 individuals.
Trombiculidae	*Walchia micropelta* (34,904); Ascoschoengastiaindica^*^ (26,193); Leptotrombidiumdeliense^*^ (21,277); *W. turmalis* (4,915); *L*.scutellare^*^ (3,948); *W. minuscuta* (1,521); *L. gongshanense* (1,486); *L. trapezoidum* (1,361); *A. yunnanensis* (1,323); *W. sheensis* (1,237); *L*.sialkotense^*^ (1,137); *Microtrombicula vitosa* (998); *L. suense* (843); *Helenicula hsui* (828); *L. eothenomydis* (771); *L*.rubellum^*^ (659); *L. longchuanense* (543); *H. miyagawai* (444); *L. muntiaci* (393); *Gahrliepia yangchenensis* (359); *L. hsui* (314); *H. yunnanensis* (312); *Doloisia moica* (284); *Blankaartia acuscutellaris* (283); *L. allosetum* (273); *W. Chuanic*a (217); *L. qiui* (191); *L. bishanense* (189); *W*.pacifica^*^ (159); *W. rustica* (143); *L. shuqui* (113); *D. sinensis* (107); *L*.imphalum^*^ (99); *W*.chinensis^*^ (89); *A. menghaiensis* (87); *L. longimedium* (79); *H. simena* (64); *L. densipunctatum* (59); *L. spicanisetum* (55); *H. lanius* (55); *D. spatulata* (53); *L. hiemalis* (50); *W. xishaensis* (50); *L. kunmingense* (49); *Schoengastiella ligula* (47); *L. bambicola* (46); *A. lorius* (43); *H. abaensis* (42); *L. rusticum* (36); *M. nadchatrami* (30); *L. yulini* (27); *W. kor* (21); *D. brachypus* (17); *L. quadrifurcatum* (16); *D. manipurensis* (14); *W. ewingi* (13); *L*.guzhangense^*^ (12); *A. crassiclava* (12); *L. dichotogalium* (11); *G. zhongwoi* (11); *A. yunwui* (10); *G. silvatica* (10); *L. chuanxi* (9); *A. petauristae* (9); *W. zangnanica* (9); *W. latiscuta* (9); *D. longensis* (8); *A. audyi* (7); *G. octosetosa* (7); *Trombiculindus yunnanus* (6); *Walchiella notiala* (6); *G. meridionalis* (6); *L. sinicum* (5); *L. xiaowei* (5); *T. hunanye* (5); *M. yanmai* (5); *H. globularis* (4); *W. acugastia* (4); *W. cordiopelta* (4); *L. cangjiangense* (3); *L. robustisetum* (3); *L*.akamushi^*^ (3); *L. kawamurai* (3); *T. cuneatus* (3); *M. munda* (3); *W. nanfangis* (3); *L*.yui^*^ (2); *L. xiaguanense* (5); *L. qujingense* (2); T. nujiange (2); *D. outoensis* (2); *L. aliena* (3); *W. yingjiangensis* (2); *G. lengshui* (2); *G. tenuiclava* (2); *L. fujianense* (1); *L. lianghense* (1); *L. biluoxueshanense* (1); *L*.apodemi^*^ (1); *L. jianshanense* (1); *T. spinifoliatus* (1); *H. kohlsi* (1); *Cheladonta deqinensis* (1); *A. montana* (1); *W. jiangxiensis* (1); *G. deqinensis* (1); *G. tenella* (1); *G. banyei* (1); *G. xiaowoi* (1).
**Gamasid mites**	A total of 2 families, 12 genera and 49 species with 33,378 individuals.
Laelapidae	*Laelaps nuttalli* (10,795); *L. liui* (6,830); *Dipolaelaps jiangkouensis* (4,401); *L*.echidninus^*^ (3,370); *L*.turkestanicus^*^ (3,054); *L. traubi* (1,757); *L. fukienensis* (1,413); *L*.algericus^*^ (742); *L. cheni* (299); *L. jinghaensis* (98); *L. chin* (75); Haemolaelapscasalis^*^ (69); *L. guizhouensis* (66); *L*.clethsionomydis^*^ (47); Tricholaelapsmyonysognathus^*^ (46); *Hypoaspis pavlo*vs*kii* (45); *L*.jettmari^*^ (42); *H. concinna* (47); *H. aculeifer* (22); *H. chelaris* (17); *H. orientalis* (19); *L. paucisetosa* (9); *H. ovatus* (99); *Hyperlaelaps amphibian* (7); *H. lubrica* (7); *Cosmolaelaps yerulyuae* (6); *Eulaelaps stabularis* (6); *L. extremi* (4); *Androlaelaps singularis* (4); Hirstionyssusisabellinus^*^ (4); *L. hongaiensis* (3); *H. cordatus* (3); *H. kirinensis* (3); *H. leeae* (3); *E. kanshuensis* (3); *E. jilinensis* (2); *E. pratentis* (2); *L. hsui* (1); *L. muris* (1); *L. taingueni* (1); *D. chimmarogalis* (1); *H*.glasgowi^*^ (1); *H. petauristae* (1); *H. hrdyi* (1); *H. linteyini* (1); *E. substabularis* (1); *Haemogamasus monticola* (1); *H*.nidi^*^ (1).
Macronyssidae	Ornithonyssusbacoti^*^ (55).
Total of two mite groups	A total of 3 families, 24 genera and 158 species with 142,471 individuals

^*^Vector mite species, which can serve as vectors or potential vectors of scrub typhus or HFRS.

Of the 158 mite species and 142,471 individuals, chiggers accounted for 68.99% (109/158) of species composition and 76.57% (109,093/142,471) of abundance, outnumbering gamasid mites with 31.01% of species (49/158) and 23.43% of abundance (33,378/142,471; Table 3).

**Table 3:** Statistics for overall infestation indices of mites on small mammal host at Jingha survey site in southern Yunnan, China (April 2016–March 2017).

**Taxa of mites**	**The number of hosts**		**The number of mites and** ***C_r_*** **(%)**		**Infestation indices of mites**		
	**Number of total hosts**	**Number of infested**	**No**.	***C**_*r*_* **(%)**	***P**_*M*_* **(%)**	***MA***	***MI***
Chiggers	2,424	2,088	109,093	76.57	86.14	45.01	52.25
Gamasid mites	2,424	1,628	33,378	23.43	67.16	13.77	20.50
Total	2,424	2,301	142,471	100.00	94.93	58.78	61.92

*C*_*r*_, Constituent ratio; *P*_*M*_, Prevalence (%); *MA*, Mean abundance (mite/host); *MI*, Mean intensity (mite/host).

**Figure 2:**
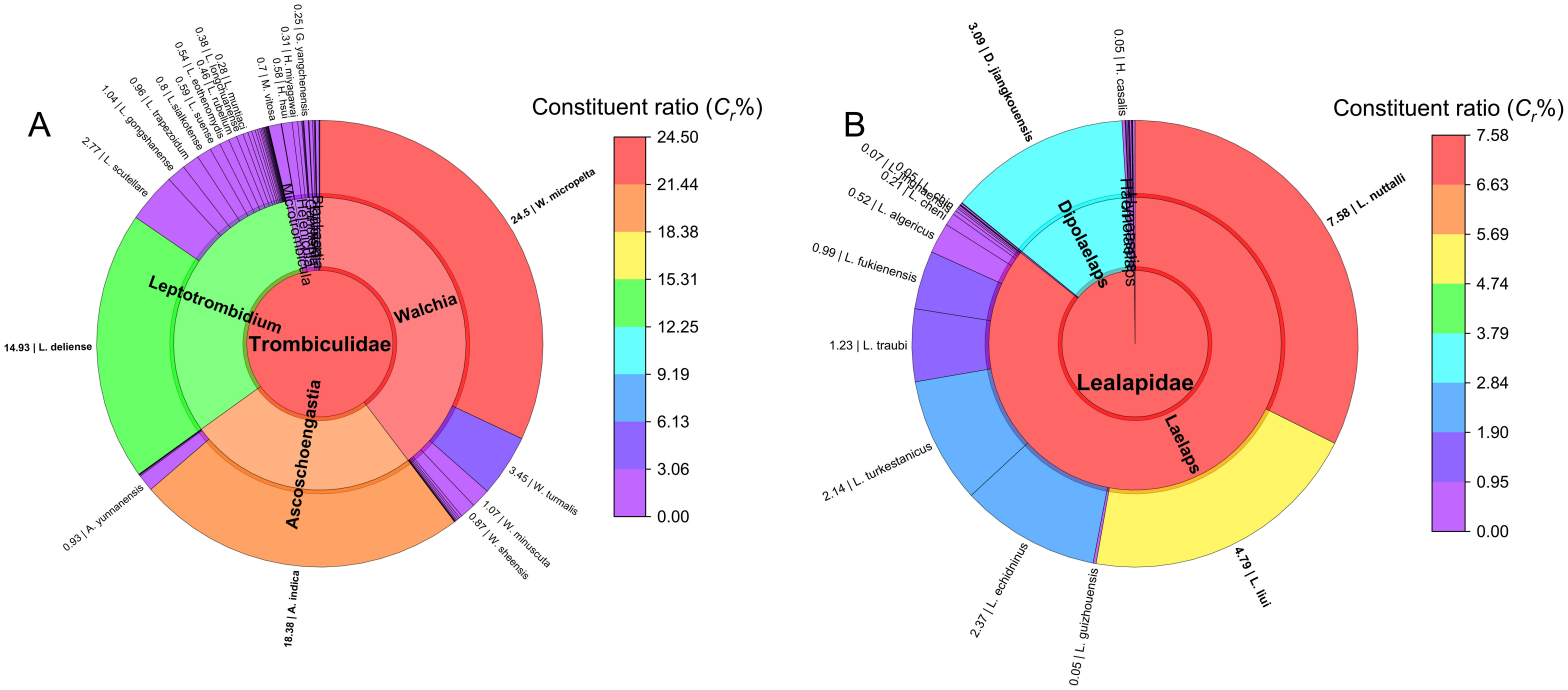
The composition of two mite groups at Jingha in southern Yunnan of southwest China (April 2016–March 2017). **(A)** The constituent ratios (*C*_*r*_) of family, genus and species of chiggers (chigger mites); **(B)** The constituent ratios (*C*_*r*_) of family, genus and species of gamasid mites.

Figure 2 illustrates the taxonomic composition of chiggers and gamasid mites at the family, genus, and species levels. At the genus level (12 genera in total), 96.31% of chigger individuals (105,065/109,093) and 73.34% of chigger species (3/12) came from three genera, *Walchia* (*C*_*r*_: 39.69% for individuals and 15.60% for species), *Ascoschoengastia* (*C*_*r*_: 25.38% for individuals and 9.17% for species), and *Leptotrombidium* (*C*_*r*_: 31.24% for individuals and 38.53% for species). The genus *Leptotrombidium* exhibited the highest species richness (42 species). At the species level (109 species in total), 75.51% of chiggers (82,374/109,093) came from three species, *W. micropelta* (*C*_*r*_ = 31.99%, 34,904/109,093), *A. indica* (*C*_*r*_ = 24.01%, 26,193/109,093), and *L. deliense* (*C*_*r*_ = 19.50%, 21,277/109,093), with *W. micropelta* showing the highest constituent ratio. Low *C*_*r*_ values ( ≤ 5%) for the remaining 106 species indicated a high proportion of rare species. Notably, *Leptotrombidium deliense* and *L. scutellare*—the primary scrub typhus vectors in China, with the latter also a potential HFRS vector—accounted for 19.50% and 3.62% of total chigger individuals, respectively.

In gamasid mite subcommunity, the family Laelapidae predominated (*C*_*r*_ = 99.84%, 33,323/33,378) at the family level (2 families). The genus-level analysis (12 genera in total) revealed the genus *Laelaps* as dominant (*C*_*r*_ = 85.71%, 28,607/33,378). Among 49 species of gamasid mites, *Laelaps nuttalli* (*C*_*r*_ = 32.34%, 10,795/33,378), *L. liui* (*C*_*r*_ = 20.46%, 6,830/33,378), and *L. echidninus* (*C*_*r*_ = 10.10%, 3,370/33,378) were the most abundant, collectively representing 62.90% of individuals (20,995/33,378; Figure 2).

Overall infestation indices revealed that chiggers exhibited significantly higher prevalence (*P*_*M*_ = 86.14%), mean abundance (*MA* = 45.01 mites per examined host), and mean intensity (*MI* = 52.25 mites per infested host) than gamasid mites (*P*_*M*_ = 67.16%, *MA* = 13.77, *MI* = 20.50). These differences were statistically significant (*P*_*M*_: χ^2^ = 243.87, *P* < 0.001; *MA*: *Z* = −30.05, *P* < 0.001; *MI*: *Z* = −27.67, *P* < 0.001; Table 3).

### 3.2 Host selection of mites

**Figure 3:**
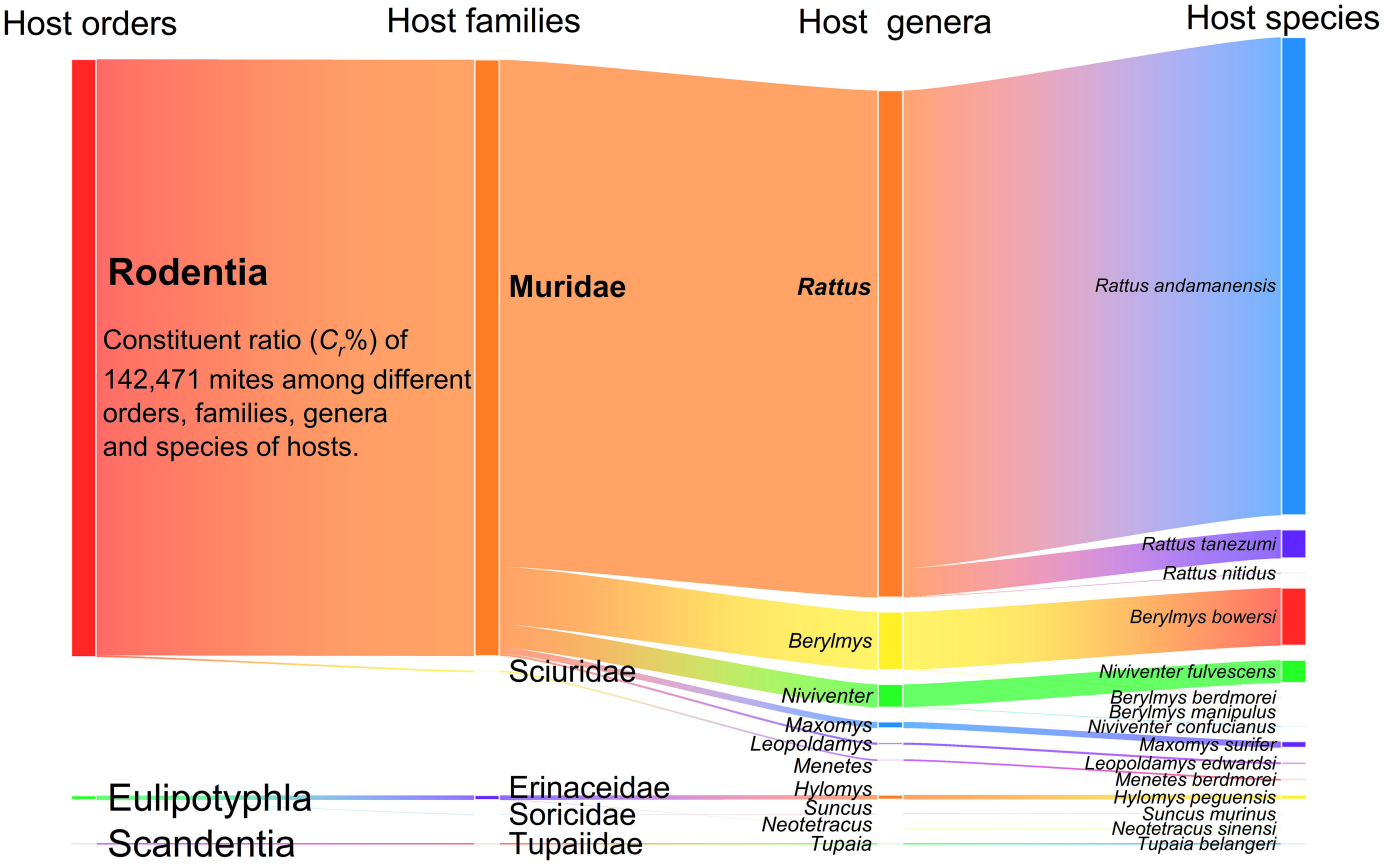
Visualization of mite distribution among different orders, families, genera and species of small mammal hosts at Jingha survey site in southern Yunnan of southwest China (April 2016-March 2017).

The results demonstrate rodents—particularly Muridae, *Rattus*, and *R. andamanensis* —constitute primary mite hosts. Sankey diagram visualization (Figure 3) of 142,471 mites across four host taxonomic levels (order, family, genus, species) revealed pronounced host preference. At the order level (*n* = 3), Rodentia harbored 99.01% of mites (*C*_*r*_ = 99.01%; 141,062/142,471). Among families (*n* = 5), Muridae hosted 98.73% of mites (*C*_*r*_ = 98.73%; 140,667/142,471). At the genus level (*n* = 10), The mites on *Rattus* predominated (*C*_*r*_ = 83.96%; 119,612/142,471), followed distantly by *Berylmys* (*C*_*r*_ = 9.58%). Species-level analysis (*n* = 15) showed 79.14% of mites infested *Rattus andamanensis* (*C*_*r*_ = 79.14; 112,752/142,471).

### 3.3 Community structure of mites and their dominant species

The mite community comprised two subcommunities: chigger and gamasid mite subcommunities. The chigger subcommunity exhibited greater species richness (109 vs. 49), Margalef index (9.31 vs. 4.61), and Shannon-Wiener diversity (2.13 vs. 1.97) than the gamasid mite subcommunity. Conversely, the gamasid mite subcommunity showed marginally higher Simpson dominance (*D* = 0.81 vs. 0.80) than the chigger subcommunity (Table 4).

**Table 4:** Community indexes of two mite groups on small mammal hosts at Jingha survey site in southern Yunnan of southwest China (April 2016–March 2017).

**Mite subcommunities**	**Indexes of mite subcommunities**				
	***S***	***R***	***H***	***E***	***D***
Chigger subcommunity	109	9.31	2.13	0.45	0.80
Gamasid mite subcommunity	49	4.61	1.97	0.51	0.81
The whole mite community	158	13.23	2.64	0.52	0.87

*S*, Species richness; *R*, Margalef richness index; *H*, Shannon-Wiener's diversity index; *E*, Pielou's evenness; *D*, Simpson's dominance index.

Four species (three from chigger subcommunity and one from gamasid subcommunity) exhibit dominance (*C*_*r*_ = 65.40%) in the combined mite community. In the chigger subcommunity, *W. micropelta* (*C*_*r*_ = 31.99%, 34,904/109,093), *A. indica* (*C*_*r*_ = 24.01%, 26,193/109,093), and *L. deliense* (*C*_*r*_ = 19.50%, 21,277/109,093) were the three dominant species. Their prevalence (*P*_*M*_) was 69.06%, 61.18%, and 62.83%, respectively, with high mean abundance (*MA* = 14.40; 10.81; 8.78) and mean intensity (*MI* = 20.85; 17.66; 13.97). In the gamasid subcommunity, *Laelaps nuttalli* was the predominant species, comprising 32.24% of all gamasid mites (*C*_*r*_ = 32.24%, 10,795/33,378), with *P*_*M*_ = of 53.42%, *MA* = 4.45, and *MI* = of 8.34. The dominating chigger species showed higher constituent ratio (*C*_*r*_) and infestation indices (*P*_*M*_, *MA, MI*) than the dominating gamasid species.

### 3.4 Vector mite species and their infestation status

We documented four primary confirmed or putative vectors of zoonotic diseases in Jingha: three chigger species (*L. deliense, A. indica, L. scutellare*) and one gamasid mite species (*Laelaps echidninus*). These species collectively accounted for 38.46% (54,788/142,471) of all the 158 identified species (109 chigger mites + 49 gamasid mites). Among these four vector mite species, *L. deliense* (*C*_*r*_ = 14.93%, 21,277/142,471) and *A. indica* (*C*_*r*_ = 18.38%, 26,193/142,471) were the most abundant with high infestation indices (Table 5).

**Table 5:** Infestation indexes of four main vector mite species at Jingha survey site in southern Yunnan of southwest China (April 2016–March 2017).

**Main vector mite species**	**No. of hosts**		**No. and constituent ratios** (***C_r_*****) of vector mites**		**Infestation indexes of vector mites**		
	**Hosts examined**	**Hosts infested**	**No**.	***C**_*r*_* **(%)**	***P**_*M*_* **(%)**	***MA***	***MI***
*Leptotrombidium deliense*	2,424	1,523	21,277	14.93	62.83	8.78	13.97
*Ascoschoengastia indica*	2,424	1,483	26,193	18.38	61.18	10.81	17.66
*Leptotrombidium scutellare*	2,424	496	3,948	2.77	20.46	1.63	7.96
*Laelaps echidninus*	2,424	779	3,370	2.37	32.14	1.39	4.33
Total	2,424	2,031	54,788	38.46	83.79	22.60	26.98

### 3.5 Seasonal dynamics of mite communities

**Figure 4:**
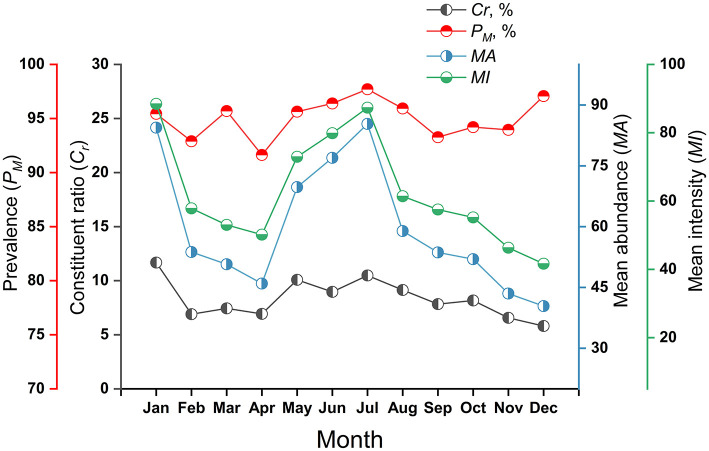
Monthly variations of mite constituent ratios (*C*_*r*_) and mite infestation indexes (*P*_*M*_, *MA, MI*) on small mammal hosts at Jingha survey site in southern Yunnan of southwest China (April 2016–March 2017).

The constituent ratios (*C*_*r*_) and overall infestation indices (*P*_*M*_, *MA, MI*) of mites on small mammal hosts fluctuated in different months. The variations of *C*_*r*_, *MA* and *MI* in different months were of statistical significance with χ^2^ = 5,884.50 and *P* < 0.001 (Chi-square test) for *C*_*r*_, *H'* = 59.25 and *P* < 0.001 for *MA* (Kruskal-Wallis *H* test), and *H'* = 56.66 and *P* < 0.001 for *MI* (Kruskal-Wallis *H* test). The difference of *P*_*M*_, however, was of no statistical significance with χ^2^ = 14.83 and *P* = 0.19 in Chi-square test (Table 6, Figure 4).

**Table 6:** Monthly variation of mite infestation indexes on small mammal host at Jingha survey site in southern Yunnan of southwest China (April 2016–March 2017).

**Month**	**The number of hosts**		**Number of mites and** ***C_r_*** **(%)**		**Indicators of total mite infestation**		
	**Number of checks**	**Number of infections**	**No**.	***C**_*r*_* **(%)**	***P**_*M*_* **(%)**	***MA***	***MI***
1	197	188	16,630	11.67	95.43	84.42	88.46
2	183	170	9,832	6.90	92.90	53.73	57.84
3	209	200	10,602	7.44	95.69	50.73	53.01
4	215	197	9,880	6.93	91.63	45.95	50.15
5	206	197	14,367	10.08	95.63	69.74	72.93
6	166	160	12,776	8.97	96.39	76.96	79.85
7	175	171	14,931	10.48	97.71	85.32	87.32
8	221	212	13,016	9.14	95.93	58.90	61.40
9	208	194	11,156	7.83	93.27	53.63	57.51
10	224	211	11,642	8.17	94.20	51.97	55.18
11	215	202	9,355	6.57	93.95	43.51	46.31
12	205	199	8,284	5.81	97.07	40.41	41.63
Total	2,424	2,301	142,471	100.00	94.93	58.78	61.92

The monthly fluctuated values of *C*_*r*_ ranged from 5.81% (December) to 11.67% (January), peaking in January. The values of *P*_*M*_ fluctuated between 91.63% (April) and 97.71% (July), with marginally elevated values in summer (July) and winter (December), but no pronounced peak. The *MA* varied from 40.41 (December) to 85.32 (July), exhibiting distinct bimodal peaks in July and December. Similarly, *MI* ranged from 41.63 (December) to 88.46 (January), with analogous bimodal peaks during July and December (Table 6, Figure 4).

**Figure 5:**
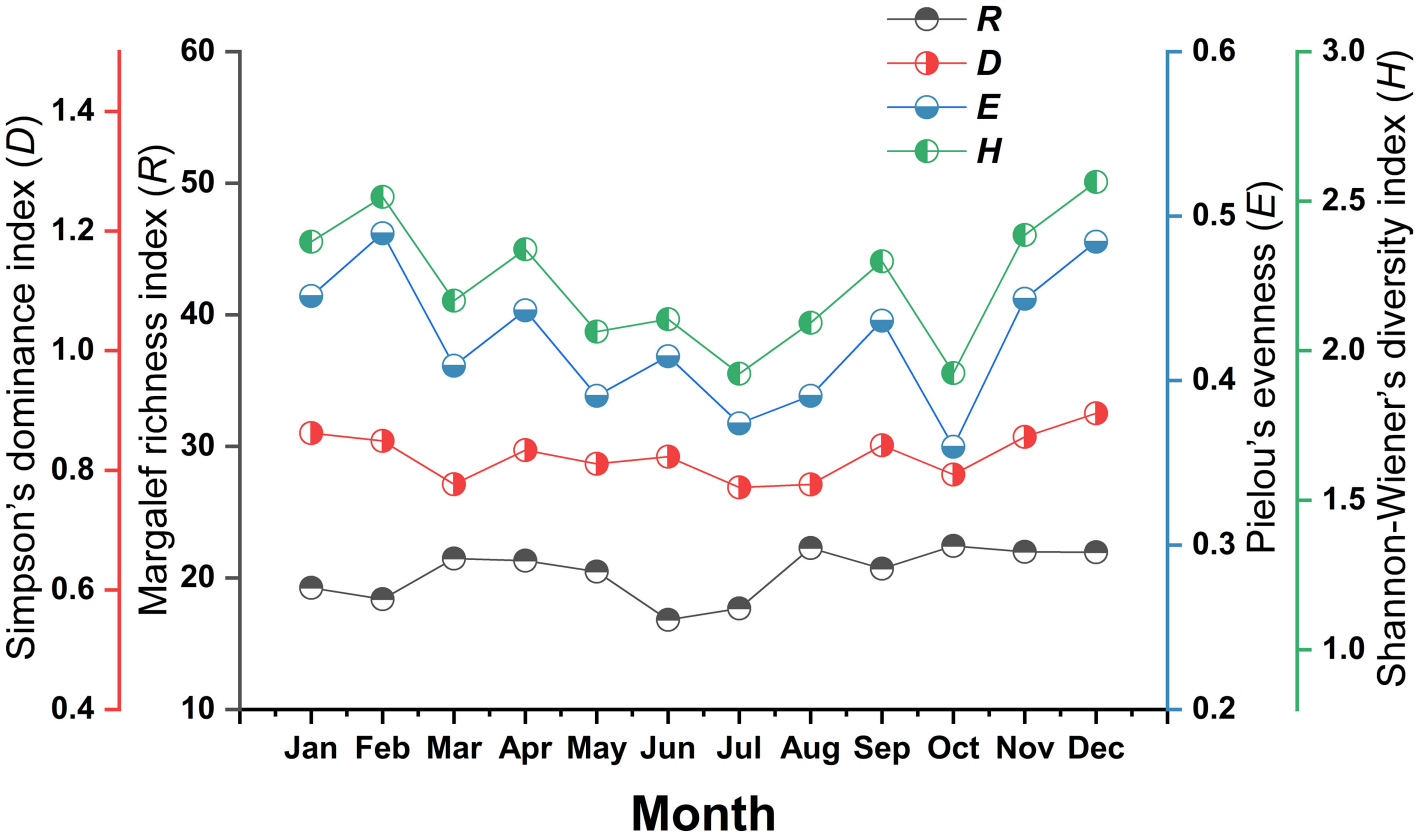
Monthly variation of mite community indexes on small mammal host at Jingha survey site in southern Yunnan of southwest China (April 2016–March 2017).

The diversity indices of the mite community in each month also showed varying degrees of fluctuations. Shannon-Wiener diversity (*H*) and Pielou's evenness (*E*) showed two minor peaks in February and December, with low ebbs occurring in October. Margalef's richness (*R*) and Simpson's dominance (*D*) slight fluctuates in different months without an obvious pattern (Figure 5).

### 3.6 Seasonal fluctuations of dominant mite species

**Figure 6:**
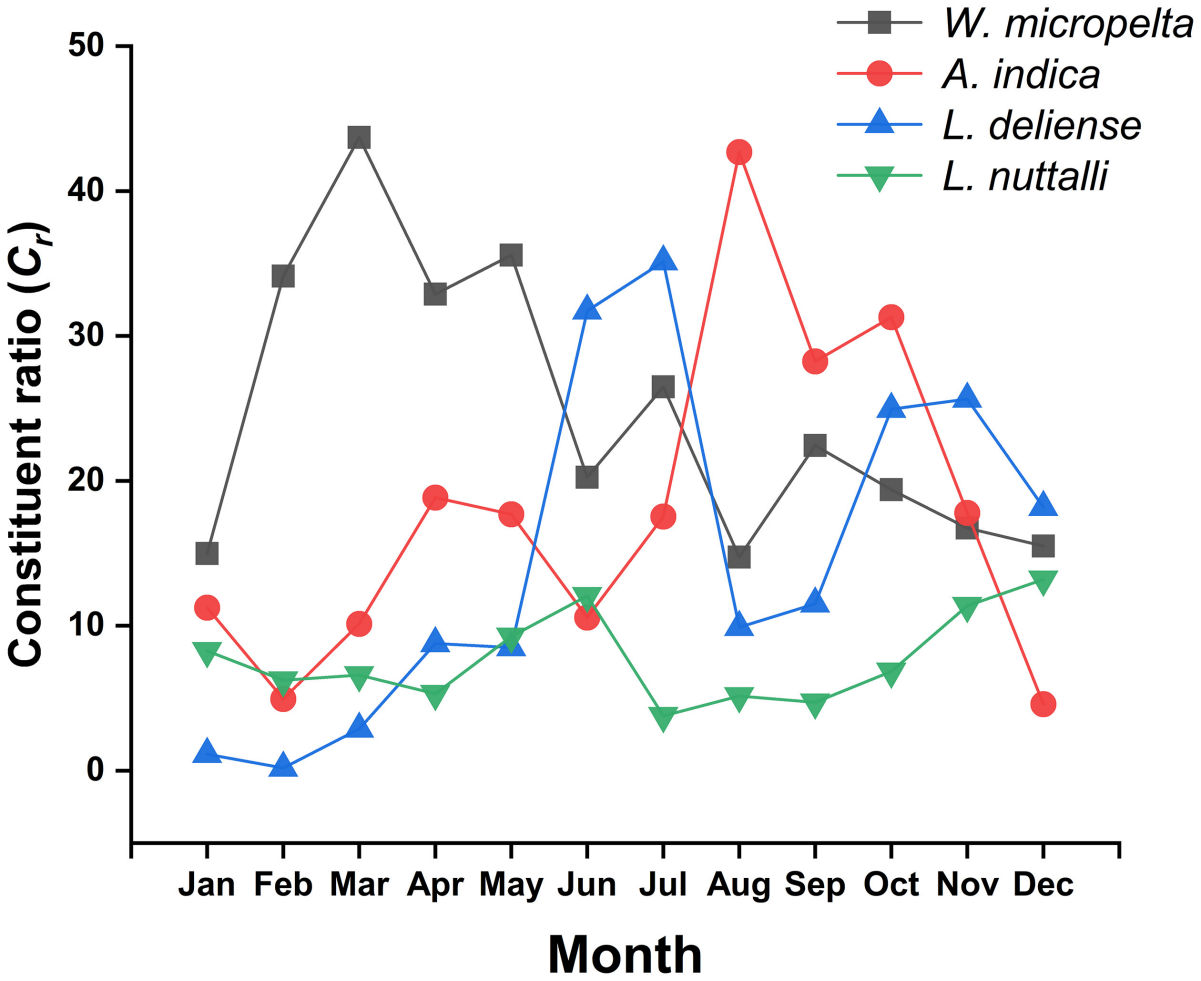
Monthly variations of constituent ratios (*C*_*r*_) of four dominant mite species at Jingha survey site in southern Yunnan of southwest China (April 2016–March 2017).

Of the dominative three chigger species (*W. micropelta, A. indica, L. deliense*) and one gamasid mite species (*L. nuttalli*), the constituent ratios (*C*_*r*_) exhibited monthly fluctuations. Specifically, *W. micropelta* peaked in early spring (March: *C*_*r*_ = 43.70%, 4,633/10,602), while *A. indica* peaked in late summer (August: *C*_*r*_ = 42.69%, 5,556/13,016). *Leptotrombidium deliense* displayed a bimodal peak distribution, with a primary peak occurring in summer (June: *C*_*r*_ = 31.73%, 4,054/12,776; July: *C*_*r*_ = 35.12%, 5,244/14,931) and a secondary peak in autumn (October: *C*_*r*_ = 24.95%, 2,905/11,642; November: *C*_*r*_ = 25.64%, 2,399/9,355). *Laelap nuttalli* exhibited minor peaks in early summer (June: *C*_*r*_ = 12.05%, 1,539/12,776) and early winter (December: *C*_*r*_ = 13.19%, 1,093/8,284; Table 7, Figure 6).

**Table 7:** Monthly variations of numbers and constituent ratios (*C*_*r*_) of four dominant mite species at Jingha survey site in southern Yunnan of southwest China (April 2016–March 2017).

**Months**	***Walchia micropelta***		***Ascoschoengastia indica***		***Leptotrombidium deliense***		***Laelaps nuttalli***	
	**No**.	***C**_*r*_* **, %**	**No**.	***C**_*r*_* **, %**	**No**.	***C**_*r*_* **, %**	**No**.	***C**_*r*_* **, %**
1	2,491	14.98	1,871	11.25	188	1.13	1,373	8.26
2	3,356	34.13	486	4.94	19	0.19	614	6.24
3	4,633	43.70	1,074	10.13	304	2.87	699	6.59
4	3,249	32.88	1,861	18.84	867	8.78	525	5.31
5	5,110	35.57	2,542	17.69	1,220	8.49	1,329	9.25
6	2,586	20.24	1,350	10.57	4,054	31.73	1,539	12.05
7	3,953	26.48	2,617	17.53	5,244	35.12	565	3.78
8	1,918	14.74	5,556	42.69	1,287	9.89	670	5.15
9	2,502	22.43	3,151	28.24	1,284	11.51	525	4.71
10	2,257	19.39	3,643	31.29	2,905	24.95	798	6.85
11	1,566	16.74	1,663	17.78	2,399	25.64	1,065	11.38
12	1,283	15.49	379	4.58	1,506	18.18	1,093	13.19
Total	34,904	24.50	26,193	18.38	21,277	14.93	10,795	7.58

### 3.7 Seasonal fluctuations of vector mite species

**Figure 7:**
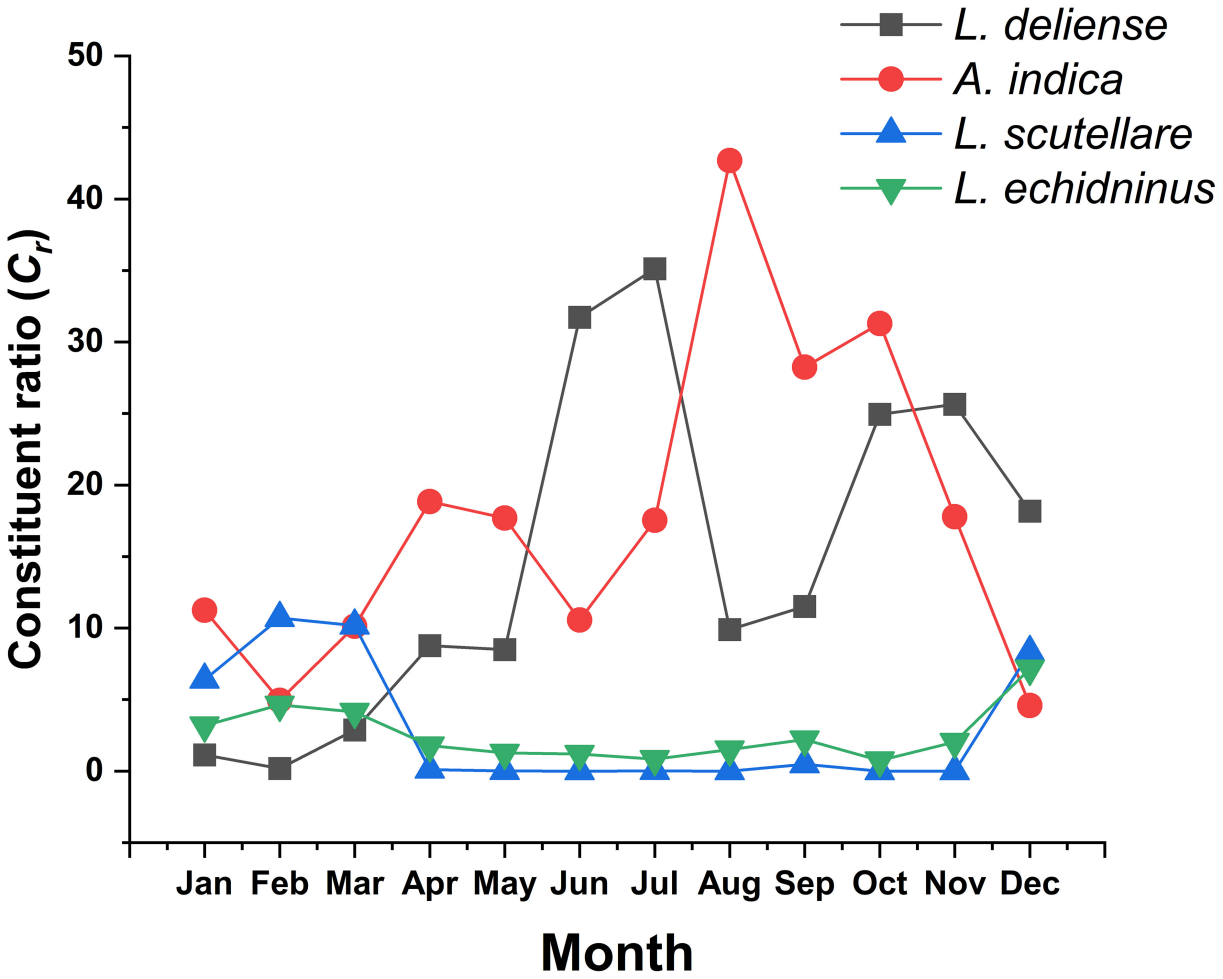
Monthly variations of constituent ratios (*C*_*r*_) of four vector mite species at Jingha survey site in southern Yunnan of southwest China (April 2016–March 2017).

The three chigger (*L. deliense, A. indica, L. scutellare*) and one gamasid (*L. echidninus*) vector species varied significantly in monthly constituent ratios (*C*_*r*_), with *L. deliense* and *A. indica* demonstrating the most substantial fluctuations. *Leptotrombidium deliense* showed bimodal peaks (summer primary peak: June-July; autumn secondary peak: October–November). *Ascoschoengastia indica* peaked in late summer (August: *C*_*r*_ = 42.69%, 5,556/13,016), while both *L. scutellare* and *L. echidninus* exhibited minor and indistinct peaks during December (Figure 7).

### 3.8 Host sex and age bias in mite infestation

**Figure 8:**
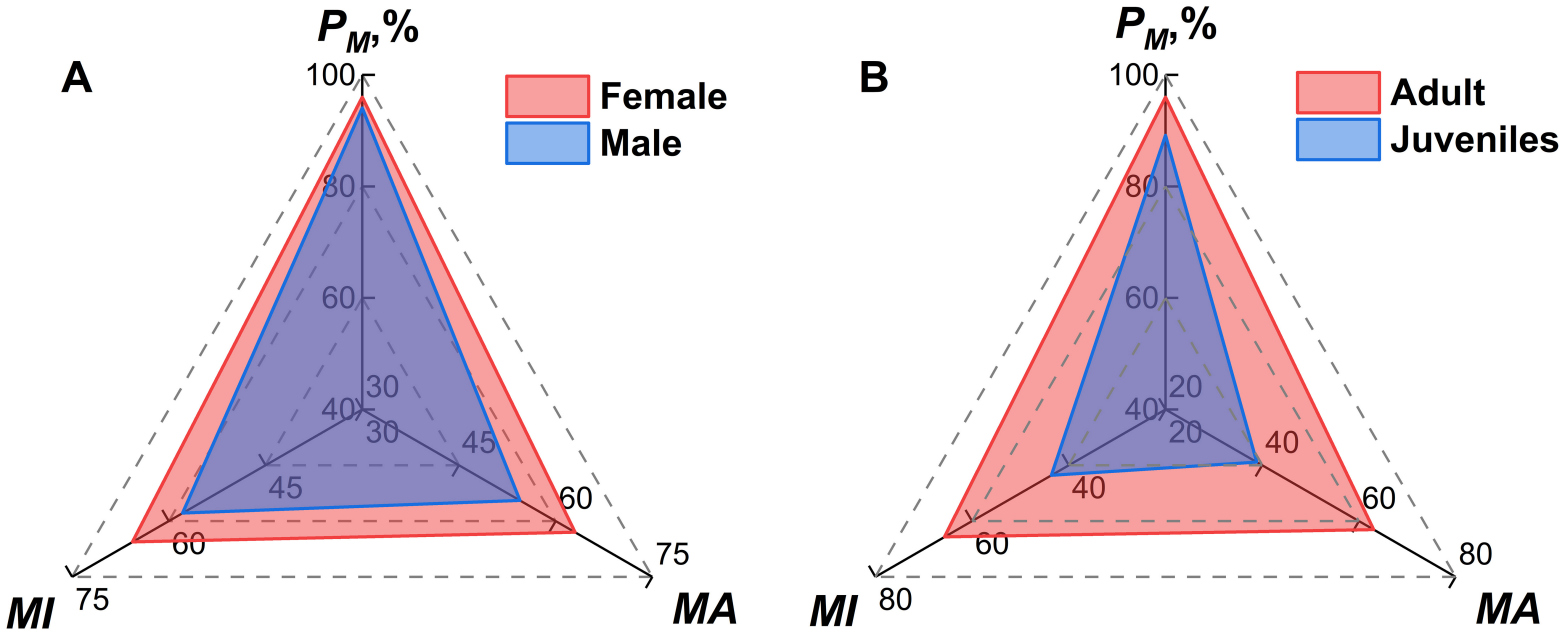
Radar chart visualization of mite infestation indexes on different sexes and ages of small mammal hosts at Jingha survey site in southern Yunnan of southwest China (April 2016–March 2017).

Host sexes and ages significantly influenced mite infestation patterns. Of the 2,424 hosts, five lacked sex data and 95 lacked age records, which were not included in the analysis of the present study. After the exclusion, the analysis included 2,419 hosts with sex records (1,253 female; 1,166 male) and 2,329 hosts with age records (1,951 adult; 378 juvenile). Female hosts exhibited significantly higher infestation indices than males (*P*_*M*_: 96.01% vs. 94.08%, χ^2^ = 4.80, *P* < 0.05; *MA*: 63.01 vs. 54.46, *Z* = −4.23, *P* < 0.001; *MI*: 65.63 vs. 57.88, Z = −3.68, *P* < 0.001; Table 8, Figure 8).

**Table 8:** Infestation indices of mites by sex and age of hosts at the Jingha survey site, southern Yunnan, China (April 2016–March 2017).

**Host sexes and ages**		**Number of hosts**	**Indexes of mite infestation**		
			***P**_*M*_* **(%)**	***MA***	***MI***
Host sexes	Female	1,253	96.01	63.01	65.63
	Male	1,166	94.08	54.46	57.88
	Total	2,419	95.08	58.89	61.94
Host ages	Adult	1,951	96.00	63.07	65.70
	Juvenile	378	89.15	38.88	43.61
	Total	2,329	94.89	59.15	62.33

Five hosts without sex records were not included in the statistics, and 95 hosts without age records were not included in the statistics.

Adult hosts showed higher infestation indices of mites than juveniles (*P*_*M*_: 96.00% vs. 89.15%, χ^2^ = 30.63, *P* < 0.001; *MA*: 63.07 vs. 38.88, *Z* = −7.71, *P* < 0.001; *MI*: 65.70 vs. 43.61, *Z* = −6.21, *P* < 0.001). All differences were statistically significant (*P* < 0.05; Figure 8).

### 3.9 Climatic drivers for the seasonal dynamics of mites

All parametric and smooth items were statistical significance (*P* < 0.001) in generalized additive models (GAMs) for the abundance of four dominant mite species: *W. micropelta, A. indica, L. deliense*, and *L. nuttall*. These outcomes demonstrating the substantial driving effect of climatic factors (monthly mean temperature, relative humidity, and precipitation) on mite abundance. The climatic driving effect on the seasonal dynamics of mites differed significantly depending on mite species. *Leptotrombidium deliense* exhibited the strongest climatic dependency with 30.6% of deviation interpretation rate (Table 9) and 73.4% of total accumulative contribution rate of climatic factors (28.1% of temperature contribution rate + 45.3% of precipitation contribution rate). *Laelap nuttalli* displayed minimal climatic responsiveness (6.47% of deviation interpretation rate).

**Table 9:** Analysis of non-linear relationships between the abundance of four dominant mite species and climate factors by using poisson-based generalized additive models (GAMs) at Jingha survey site in southern Yunnan of southwest China (April 2016–March 2017).

**Name of mite species**	***R* ^2^_adj_**	**Deviation interpretation rate (%)**	**Temperature (*Chi*^2^)**	**Relative humidity (*Chi*^2^)**	**Precipitation (*Chi*^2^)**
*Walchia micropelta*	0.031	7.47	570.4^*^	2,717.3^*^	710.1^*^
*Ascoschoengastia indica*	0.057	13.6	838.74^*^	33.33^*^	2,242.9^*^
*Leptotrombidium deliense*	0.112	30.6	834.3^*^	592.2^*^	2,047.2^*^
*Laelaps nuttalli*	0.026	6.47	139.9^*^	452.9^*^	803.7^*^

The mark “^*^” indicates *p* < 0.001; Chi^2^ values reflect the importance ranking of variables.

**Figure 9:**
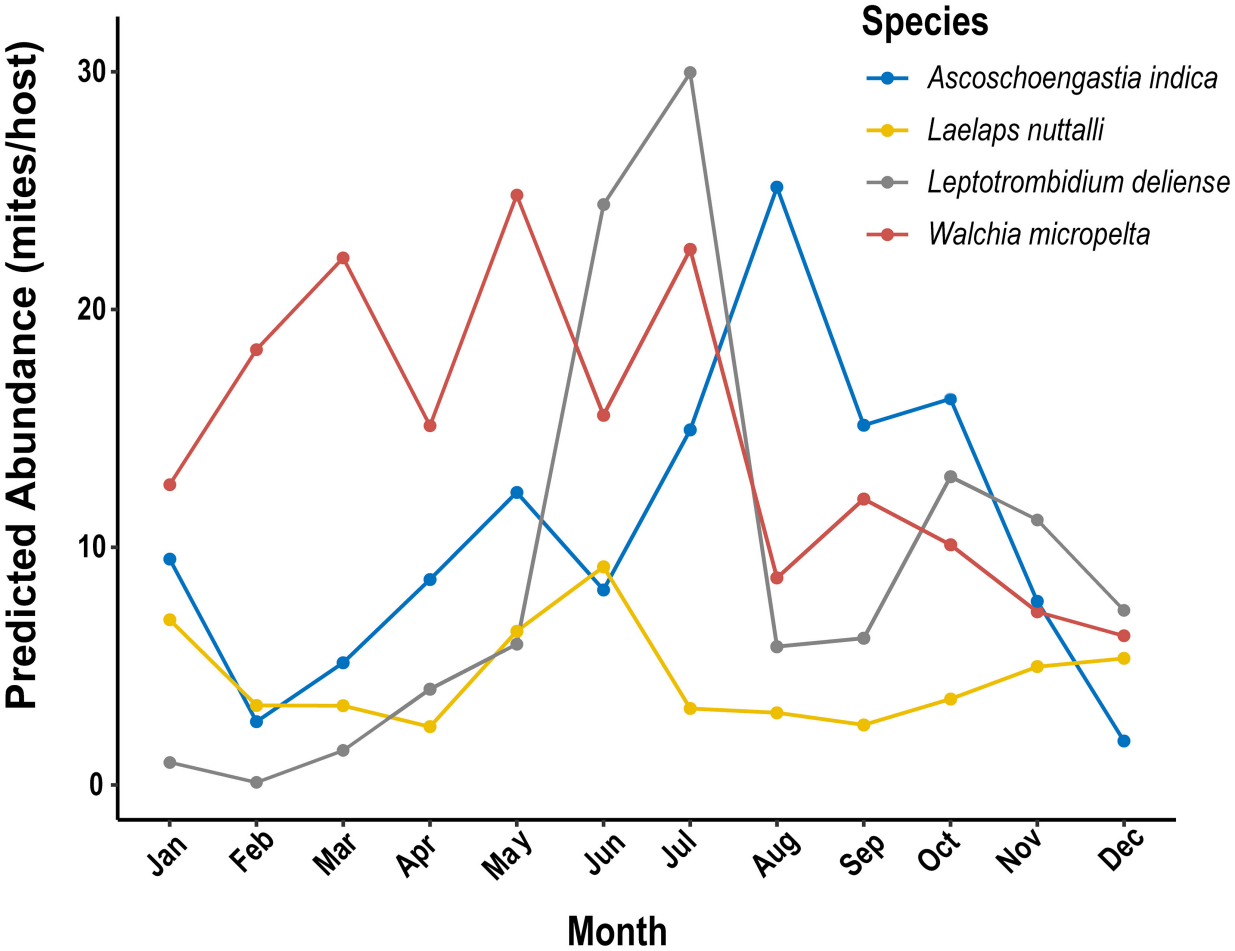
Monthly dynamics of predicted abundance of four dominant mite species driven by climate factors (temperature, relative humidity and precipitation) by using generalized additive models (GAMs) at Jingha survey site in southern Yunnan of southwest China (April 2016–March 2017).

Kruskal-Wallis tests confirmed the temporal niche differentiation of four dominant mite species (*W. micropelta, A. indica, L. deliense*, and *L. nuttalli*) at Jingha site (χ^2^ = 38.70, *P* < 0.001), with four types of climatic responses as follows (Figure 9).

(1) Multimodal temperature-response type: The abundance of *W. micropelta* peaked in March (22.2 mites/host; 95% CI: 21.5–22.8), May (24.8 mites/host; 95% CI: 24.1–25.5) and July (22.5 mites/host; 95% CI: 21.8–23.2).

(2) Precipitation-lagged response type: the abundance of *A. indica* peaked in August (25.1 mites/host; 95% CI: 24.5–25.8), which was 1 month lagged behind the maximum precipitation at Jingha site in July. The confidence interval (95% CI) for the abundance of *A. indica* did not overlap with that of *L. nuttalli* (3.0 mites/host; 95% CI: 2.8–3.3), with an obvious temporal niche differentiation (*P* < 0.01).

(3) High temperature strategic type: the abundance of *L. deliense* showed an outbreak increase in summer with high temperature and relative humidity, and its highest peak appeared in July (30.0 mites/host; 95% CI: 29.2–30.8). *Leptotrombidium deliense* showed a high adaptability to high temperature and relative humidity with the thermal tolerance of 12 °C−32 °C (mean *edf* =3.66 ± 0.12).

(4) Broad climate-adaptive type: in 12 months of a year, *L. nuttalli* showed an inapparent seasonal fluctuation with two minor peaks of mite abundance in January (7.0 mites/host) and June (9.2 mites/host), and the confidence intervals was broad (Levene's test: *F* = 7.32, *P* = 0.002), indicating that *L. nuttalli* has a broad adaptability to different types of climates throughout the year.

**Figure 10:**
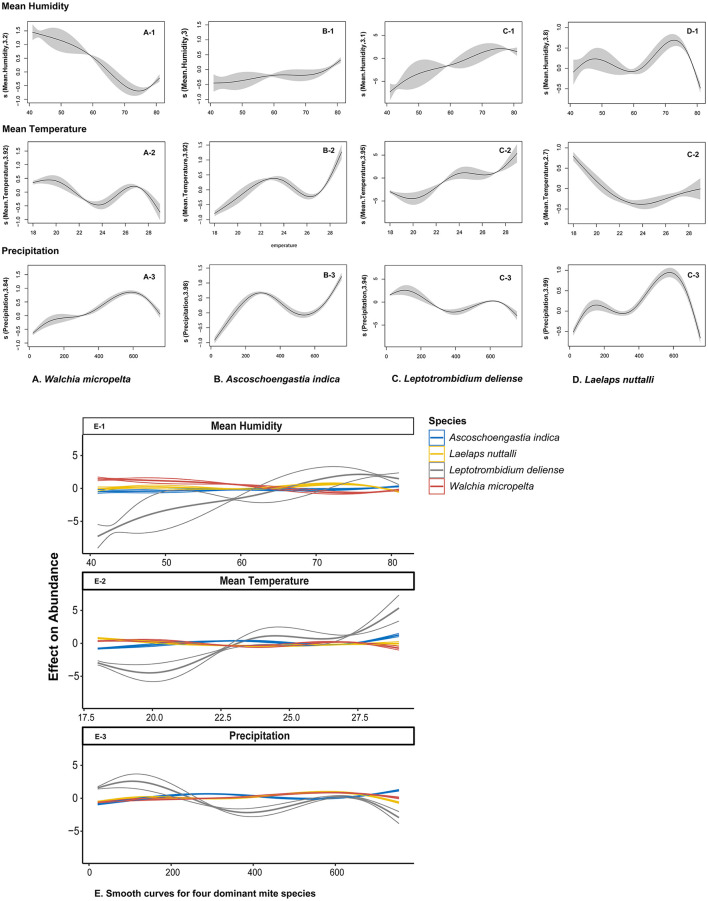
Non-linear effect of climate factors on mite abundance of four dominant mite species at Jingha survey site in southern Yunnan of southwest China (April 2016–March 2017). The curves were fitted with generalized additive models (GAMs), in which the shaded area represents 95% confidence intervals.

GAMs further revealed the response heterogeneity of different mite species to climates. The abundance of *A. indica* showed an exponential growth with the increase of precipitation and reached 25.1 mites/host (95% CI: 24.5–25.8) when the precipitation was >150 mm/month, which was 7.4 times that in the dry season when the precipitation was <50 mm. *Leptotrombidium deliense* showed a strong adaptability to the high temperature and humidity in summer with the highest peak of mite abundance in July. *Laelpas nuttalli*, however, had an inapparent seasonal fluctuation of mite abundance, and it showed a strong tolerance to different types of climates all the year round (Figures 9, 10).

### 3.10 Mite-mite network analysis

**Figure 11:**
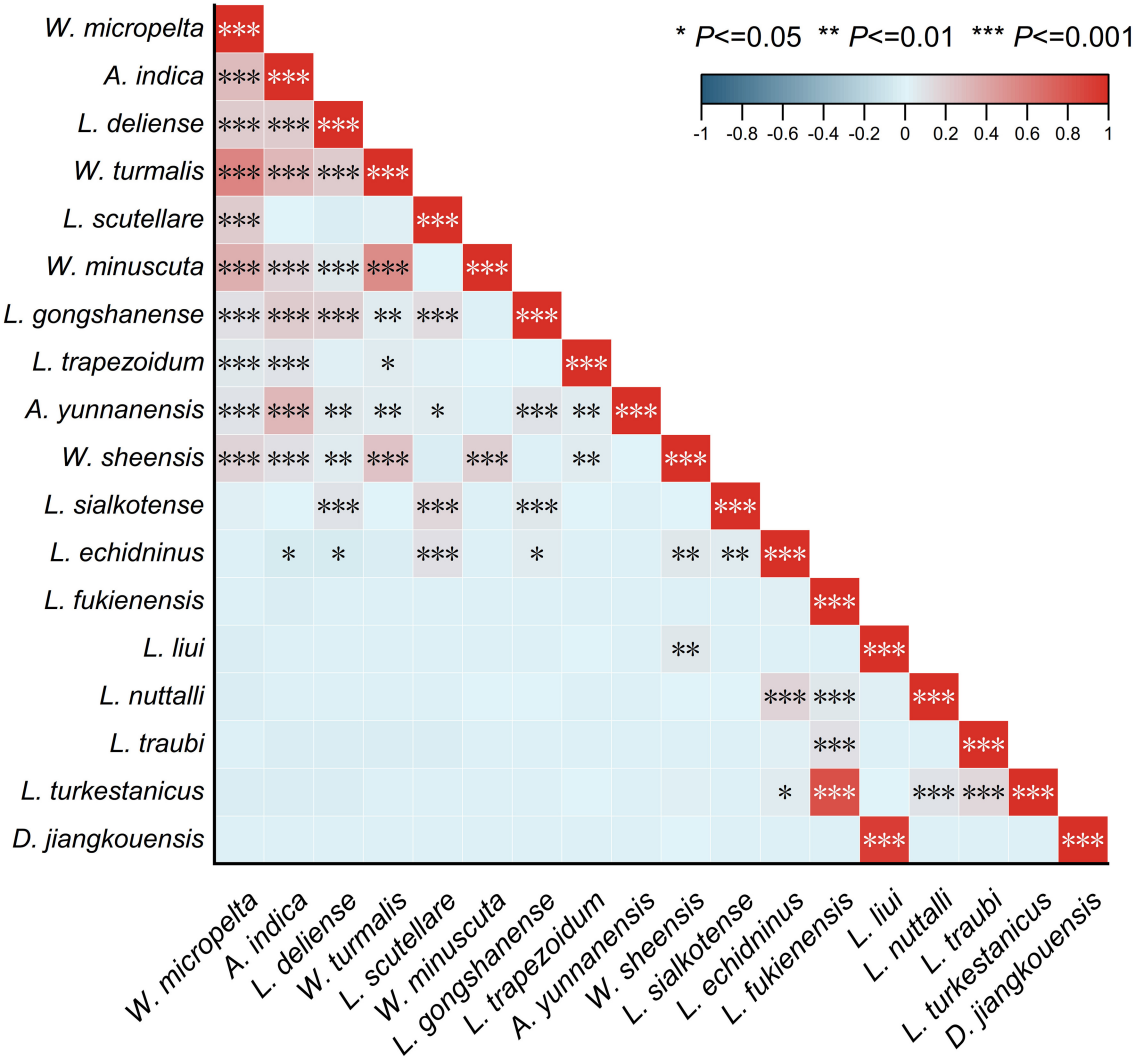
Heat map visualization for the relationship between any two of 18 main mite species at Jingha survey site in southern Yunnan of southwest China (April 2016–March 2017).

The Pearson coefficient heatmap, indicating linear correlations between species pairs (mite-mite networks), comprises 18 main mite species (individuals >1,000, accounted for 72.87% of total mites, 103,821/142,471; Figure 11). Among the resulting 153 species-pairs, 32.68% of which (50/153) showed varying degrees of correlation with statistical significance (*P* < 0.05). Positive correlations mainly occurred within the same mite group (chigger–chigger or gamasid–gamasid mites), which accounted for 96.00% of all the correlated species-pairs (48/50). For example, a high positive correlation existed between two pairs of gamasid mites with correlation coefficients *r* = 0.94 for *Dipolaelaps jiangkouensis* vs. *L. liui* (*P* < 0.001) and *r* = 0.82 for *L. turkestanicus* vs. *L. fukienensis* (*P* < 0.001). The moderate and low positive correlations mainly occurred within chigger species (*r*: 0.40–0.59 for moderate correlations; *r*: 0.20–0.39 for low correlations) such as *W. micropelta* vs. *W. turmalis* (*r* = 0.57, *P* < 0.001), *A. indica* vs. *A. yunnanensis* (*r* = 0.32, *P* < 0.001), *A. indica* vs. *W. turmalis* (*r* = 0.31, *P* < 0.001), *A. indica* vs. *L. gongshanense* (*r* = 0.21, *P* < 0.001), and *L. deliense* vs. *W. turmalis*/*W. micropelta*/*A. indica* (*r* = 0.21 for all, *P* < 0.001). There was almost no correlation between chiggers and gamasid mites, as indicated by correlation coefficients (*r*) being very close to zero (0). For example, *r* = −0.02 (*P* < 0.05) for the chigger *L. deliense* and gamasid mite *L. echidninus* (Figure 11).

### 3.11 Host-mite network analysis

**Figure 12:**
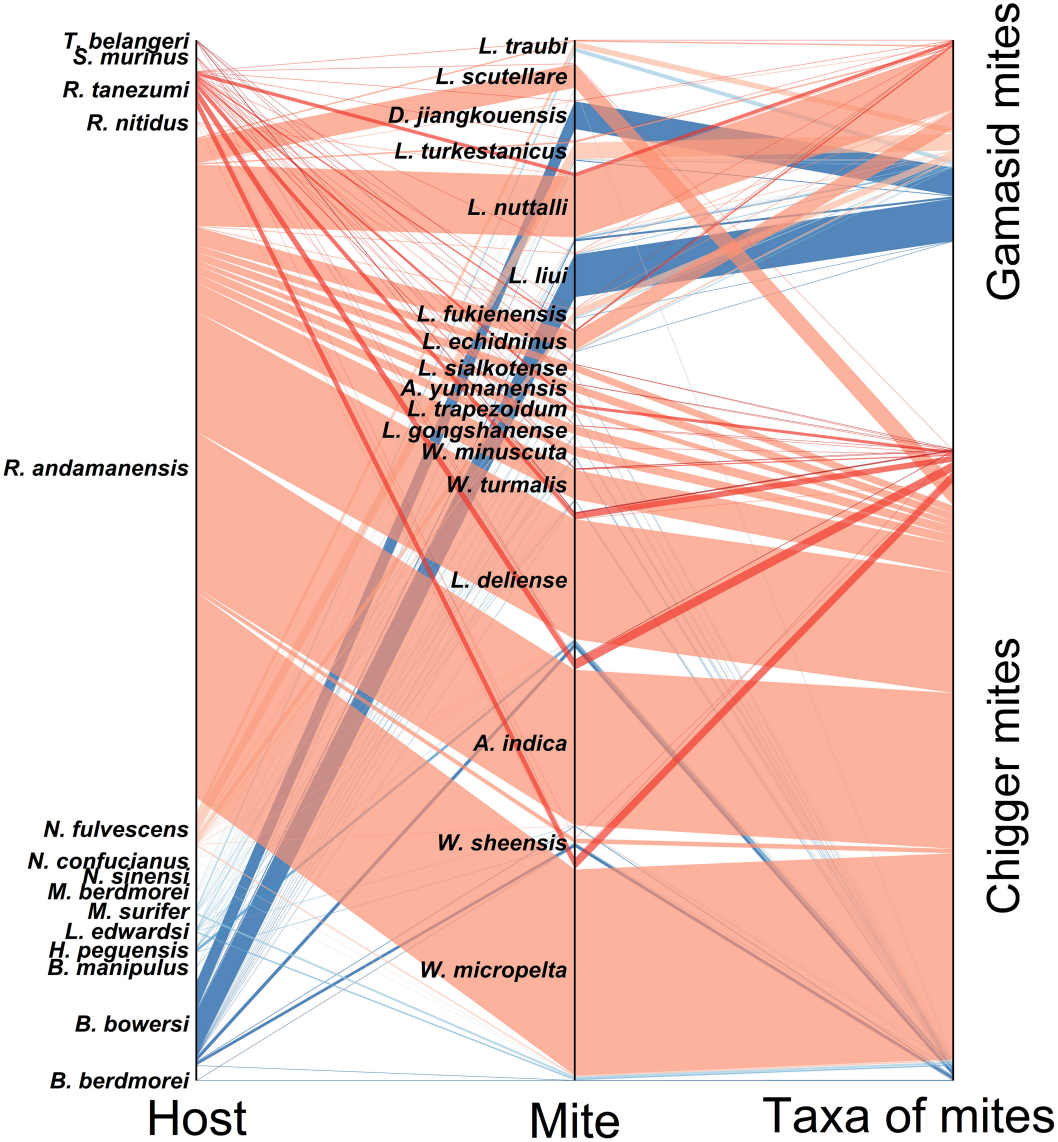
Parallel set map visualization for mutual relationships among main mite species and their small mammal hosts at Jingha survey site in southern Yunnan of southwest China (April 2016–March 2017).

The analysis of bilateral relationships was conducted between the 18 main mite species and their corresponding small mammal hosts (host-mite networks), and the results was visualized with the parallel sets diagram (Figure 12). Of the 18 main mite species, *W. micropelta* (32,395), *A. indica* (24,490) and *L. deliense* (18,867) were the most abundant. The 18 main mite species parasitized 15 small mammal species, of which *R. andamanensis* was predominant (*C*_*r*_ = 84.69%, 2,053/2,424). The results showed that one host species could harbor up to 2–18 mite species and one mite species could parasitize up to 3–11 host species with low host specificity. For example, 6,669 of *L. liui* gamasid mites were found on 12 host species, 34,904 of *W. micropelta* chiggers appeared on 11 host species, and 4,375 of *D. jiangkouensis* gamasid mites occurred on 9 host species. The host specificity of the gamasid mite *D. jiangkouensis*, however, seemed relatively high, and 4,401 of *D. jiangkouensis* mites came from only three host species, *B. bowersi, R. andamanensis*, and *R. tanezumi* (Figure 12).

## 4 Discussion

### 4.1 Mite infestation and characteristics of mite communities

Compares to previous reports (6, 8, 12, 13, 15–17, 22, 26–29), mite infestation in Jingha was relatively high, considering almost all small mammals were parasitized and the great numbers of mite individual and species collected. This pattern is comparative to other low-latitude and low-altitude regions in southern China, where warm and humid climates facilitate ectoparasites' survival and reproduction (48, 49). Previous studies in subtropical monsoon areas also reported high infestation level for chigger and gamasid mites (17, 22, 27). However, our results, especially for chiggers, are among the highest recorded (29, 34, 50). This may be due to both the favorable climate and the low host specificity. Compared with surveys in other parts of Yunnan, the chigger mite community in Jingha has higher species richness and yielded higher diversity metrics, while gamasid mite's diversity is comparatively lower. *Rattus andamanensis* is the primary host, supporting diverse mite communities and sustaining zoonotic disease cycles in similar habitats across Southeast Asia (50, 51). The dominance of chiggers over gamasid mites in both number and infestation indices implies their main role in disease transmission in this area.

### 4.2 Dominant and vector mite species

Several confirmed and potential vector species were found in Jingha, including *L. deliense, A. indica, L. scutellare*, and *L. echidninus* (Table 2). Importantly, *A. indica* and *L. deliense* were both dominant mites and key vectors (Tables 5, 10). Similar groups of species have been linked to persistent foci of scrub typhus and HFRS in Guangxi, Hainan, and northern Vietnam (52–55), highlighting the need for extra attention. *Leptotrombidium deliense* and *A. indica* are both prevalent and epidemiologically important. Their overlapping activity periods may increase the risk of pathogen spillover. *Leptotrombidium scutellare*, although less common in Jingha, is an important vector in cooler areas from central China to the north. Its presence in Jingha suggests a broader environmental tolerance than previously recognized. *Laelaps echidninus* is a widely distributed gamasid mite and has been linked to HFRS in several provinces, supporting the view that gamasid mites act as secondary but persistent vectors (17, 54, 55). The combination of high species diversity, low host specificity, and vector dominance in Jingha may raise the long-term risk of zoonotic disease transmission, necessitating increased surveillance efforts.

**Table 10:** Infestation indexes of four dominant mite species at Jingha survey site in southern Yunnan of southwest China (April 2016–March 2017).

**Dominant mite species**	**No. of hosts**		**No. and constituent ratios** (***C_r_*****) of mites**		**Infestation indexes of mites**		
	**Hosts examined**	**Hosts infested**	**No**.	***C**_*r*_* **(%)**	***P**_*M*_* **(%)**	***MA***	***MI***
*Walchia micropelta*	2,424	1,674	34,904	24.50	69.06	14.40	20.85
*Ascoschoengastia indica*	2,424	1,483	26,193	18.38	61.18	10.81	17.66
*Leptotrombidium deliense*	2,424	1,523	21,277	14.93	62.83	8.78	13.97
*Laelaps nuttalli*	2,424	1,295	10,795	7.58	53.42	4.45	8.34

### 4.3 Seasonal dynamics and driving factors

Previous studies have posited that the seasonal fluctuation patterns of chiggers and gamasid mites vary with mite species and geographical regions, i.e., different mite species exhibit different seasonal fluctuation patterns peaked in different seasons (12, 27). Moreover, the same mite species may show geographical variations in seasonal trends and peak timing (27, 35, 55). The seasonal changes of dominant mite species in Jingha show a mix of climate-specialist and climate-generalist patterns. This agrees with reports from other subtropical areas (7, 12, 22, 56).

*Leptotrombidium deliense* dually peaks in summer and autumn, similar to those reported in Guangxi, Guangdong, Taiwan and northern Thailand, which may attribute to that high temperature and humidity increase reproduction (57–62). *Ascoschoengastia indica* peaked in late summer in August, conforming to the “summer-autumn” pattern. This species displayed “rainfall-lag response” characteristics with precipitation being its primary climatic driver. *Walchia micropelta* peaked in early spring, which is different from the summer–autumn peaks of most other vector mites. This separation of peak activity may reduce competition between species and help stabilize populations (63, 64). The ecology and vector role of *W. micropelta* are not well studied, and further research is needed. *Laelaps nuttalli* and *L. echidninus* had stable populations throughout the year, reflecting broad climate tolerance. This stability allows them to act as constant pathogen reservoirs, complementing the seasonal risk from chiggers (8, 65). The observed patterns indicate that preventive practice for scrub typhus and HFRS in southern Yunnan should concentrate on summer and autumn, the peak activity periods of vectors, yet remain ongoing throughout the year due to the resilience of climate-tolerant gamasid mites.

### 4.4 Host sex and age biases in mite infestation

In this study, the mite infestation indices (*P*_*M*_, *MA, MI*) were significantly different on different sexes and ages of small mammal hosts, indicating the sex and age biases of hosts to mite infestation. Female hosts showed significantly higher infestation indices than males. Adult hosts also demonstrated markedly higher infestation levels than juveniles. Previous studies report that host sexes and ages commonly influence susceptibility to both internal and external parasites. Some research indicates that males exhibit higher parasite vulnerability than females, potentially due to males' high androgen levels, energy-intensive mating behaviors, and male-male competition (66–68), despite that greater female susceptibility to parasite infections were also reported (69–71). The significantly higher infestation in adults aligns with previous studies. This age bias pattern likely arises from adults' larger body surface area available for parasites and their expanded activity ranges during foraging and mating, increasing exposure opportunities (28, 33, 72, 73).

### 4.5 Mite-mite and host-mite relationships

Regarding host selection, different degrees of positive/negative correlations existed among 18 dominant mite species. Positive correlations predominantly occurred within gamasid or chigger subcommunities. The correlations within chigger mites were frequent but weak, while the correlations within gamasid mites were comparatively stronger (*r* > 0.60). This indicates coexisting tendencies within mite groups when colonizing hosts, aligning with prior reports on these taxa (28, 74–76). Among the 153 species pairs, only two showed negative correlations, while correlations between chiggers and gamasid mites were extremely weak. This suggests that chiggers and gamasid mites independently choose their hosts, showing neither mutual attraction nor repulsion.

The host-mite relationships demonstrate the low host specificity of mites. A certain host species can harbor multiple mite species, and a single mite species can parasitize diverse hosts. The broad host range enables mites to circulate zoonotic pathogens (e.g., *Orientia tsutsugamushi* for scrub typhus, hantavirus for HFRS) across different animal hosts. Consequently, it may increase the potential risk of persistently maintaining the endemic foci of zoonotic diseases.

## 5 Conclusion

The ectoparasitic mite community on small mammals in Jingha on the bordering zone of China and Myanmar comprises diverse gamasid and chigger mites with high species diversity, particularly pronounced in chigger mites. Both chiggers and gamasid mites have a wide range of small mammal hosts with low host specificity, and the rat *R. andamanensis* (Rodentia: Muridae) is the primary host species. Chigger mites exhibit significantly higher infestation intensity than gamasid mites. Adult hosts show greater infestation than juveniles. The dominant mite species include *W. micropelta, A. indica, L. deliense*, and *L. nuttalli*. Multiple vector mite species coexist in Jingha and the four main vector species include three chigger mites (*L. deliense, A. indica, L. scutellare*) and one gamasid mite (*L. echidninus*). Three dominant vectors (*W. micropelta, A. indica, L. deliense*) display markedly seasonal variations. Climatic factors (temperature, humidity, rainfall) drive the seasonal dynamics of mites. Abundance of *L. deliense*, one of the primary scrub typhus vectors in China, peaks in July of summer, with temperature being its main driver. *Ascoschoengastia indica*, a potential vector of scrub typhus, has 1-month lag in response to peak rainfall in July, and the precipitation mainly drives its seasonal dynamics. The summer-autumn seasonal pattern of main vector mites suggests that the surveillance of scrub typhus and HFRS in southern Yunnan should be prioritized in summer and autumn.

## Data Availability

The original contributions presented in the study are included in the article/supplementary material, further inquiries can be directed to the corresponding author.
